# A novel AP-1/miR-101 regulatory feedback loop and its implication in the migration and invasion of hepatoma cells

**DOI:** 10.1093/nar/gku872

**Published:** 2014-09-26

**Authors:** Jing-Jing Liu, Xue-Jia Lin, Xiao-Jing Yang, Liangji Zhou, Shuai He, Shi-Mei Zhuang, Jine Yang

**Affiliations:** Key Laboratory of Gene Engineering of the Ministry of Education, State Key Laboratory of Biocontrol, School of Life Sciences, Sun Yat-sen University, Guangzhou 510275, P.R. China

## Abstract

MicroRNA-101 (miR-101) is frequently downregulated in various cancers. To date, the regulatory networks of miR-101 remain obscure. In this study, we demonstrated that miR-101 was mainly transcribed from human *miR-101-2* and mouse *miR-101b*gene loci. Subsequent analyses revealed that activator protein-1 (AP-1) directly binded to the −17.4 to −16.4 k region upstream of pre-miR-101-2 and activated the expression of miR-101. On the other hand, miR-101 could inhibit the expression of ERK2 and c-Fos, two key factors of the AP-1 pathway, by binding to their 3′-UTRs. Furthermore, reintroduction of miR-101 efficiently suppressed the AP-1 activity and pri-miR-101-2 transcription. These data thus suggest a novel AP-1/miR-101 regulatory circuitry, that is, AP-1 promotes the transcription of miR-101, whereas the expression of miR-101 reduces the level of ERK2 and c-Fos and thereby attenuates the AP-1 signaling. Further investigation disclosed that the AP-1 activator TPA-induced MMP9 activity and the TPA-promoted migration and invasion of hepatoma cells were significantly attenuated by miR-101 but were enhanced by miR-101 inhibitor. Our results suggest that the AP-1/miR-101 feedback loop may prevent the excessive activation of metastatic signals imposed by ERK2/AP-1 and highlight the biological significance of miR-101 downregulation in cancer metastasis.

## INTRODUCTION

MicroRNAs (miRNAs) are small non-coding RNAs that repress protein expression at the post-transcriptional level by binding to the 3′-untranslated region (3′-UTR) of mRNAs (1). Extensive studies have revealed that miRNAs are important regulators for the maintenance of fundamental cellular processes, such as cell proliferation, differentiation and apoptosis (2). Moreover, aberrant expression of miRNAs has been found in a variety of malignancies. Accumulating evidence suggests that miRNAs may function as a novel class of oncogenes or tumor suppressor genes (3).

Deregulation of microRNA-101 (miR-101) has been implicated in the development of different malignancies (4–9). We have previously shown that miR-101 is significantly downregulated in hepatocellular carcinoma (HCC) cells, and the restoration of miR-101 dramatically promotes the apoptosis and suppresses the tumorigenicity of hepatoma cells *in vitro* and *in vivo* (6). Studies from others also disclose that miR-101 not only inhibits the proliferation and colony formation of different cancer cells (10,11) but also suppresses tumor cell migration, invasion and metastasis (4,5,7–10). These data suggest a potential tumor suppressive role of miR-101. Several molecules, including enhancer of *zeste homolog 2* (*EZH2*), *myeloid cell leukemia sequence 1* (*Mcl-1*), *c-Fos* and *MYCN*, have been experimentally validated as the targets of miR-101 (5,6,8,11). Although the tumor suppressive function of miR-101 has been largely explored, the transcriptional regulation and the regulatory network of miR-101 remain obscure.

Activator protein-1 (AP-1) is an inducible transcription factor complex that consists of a group of transcription factors including the Jun, Fos and ATF family proteins. External stimuli are transmitted to AP-1 mainly through mitogen-activated protein kinases (MAPKs) cascade (12,13). Extracellular signal regulated kinases (ERKs) 1 and 2, p38 kinases and c-Jun N-terminal kinases (JNKs) are the well-characterized subfamilies of MAPKs. Among them ERK1 and ERK2 are widely expressed and are involved in the regulation of critical cellular functions, including proliferation, differentiation, migration and apoptosis (14). When activated by upstream MAPK kinase, ERK1/2 translocate into the nucleus, where they further activate the transcription factors including AP-1. The activated AP-1 binds preferentially to the 12-*O*-tetradecanoylphorbol-13-acetate (TPA) responsive element in the gene promoter or enhancer and thereby modulates expression of target genes, like several *matrix metalloproteinases* (*MMPs*) and *CD44* (15,16).

In an attempt to explore the regulatory networks of miR-101, we revealed that AP-1 directly transactivated miR-101 and there existed a novel AP-1/miR-101 negative regulatory circuitry in hepatoma cells, whose disturbance enhanced the activity of MMP9 and thus promoted the migration and invasion of hepatoma cells. Our results highlight the importance of AP-1/miR-101 regulatory loop in preventing the abnormal transcription of pro-metastatic genes and in maintaining cellular homeostasis.

## MATERIALS AND METHODS

### Tissues

Mouse tissues were collected from 6–8-week-old C57BL/6 mice. All animal use procedures were in accordance with the Guide for the Care and Use of Laboratory Animals (NIH publications no. 80–23, revised 1996) and were performed according to the institutional ethical guidelines for animal experiments. Normal human liver tissue (661N) was collected from an individual undergoing resection of hepatic hemangiomas. Informed consent was obtained from the patient. All tissue samples were snap-frozen with liquid nitrogen and stored in a liquid nitrogen tank until use. This study was approved by the Institute Research Ethics Committee at Cancer Center, Sun Yat-sen University.

### Cell culture and transfection

Human HCC cell lines HepG2 (ATCC), Huh7, MHCC-97H, QGY-7703, SMMC-7721 and human embryonic kidney HEK293T cell were grown in Dulbecco's modified Eagle's medium (DMEM) supplemented with 10% fetal bovine serum (FBS; HyClone, Thermo Fisher Scientific, Austria), 100 U/ml penicillin and 100 μg/ml streptomycin in a humidified atmosphere of 5% CO_2_ at 37°C.

RNA oligoribonucleotides were reversely transfected using Lipofectamine RNAiMAX (Invitrogen, Carlsbad, CA, USA). A final concentration of 50 nM RNA duplex or 200 nM miRNA inhibitors was used for each transfection, unless otherwise indicated. Co-transfection of the RNA duplex and plasmid DNA was conducted using Lipofectamine 2000 (Invitrogen, Carlsbad, CA, USA). All transfections were performed according to the manufacturer's protocol.

### RNA oligoribonucleotides and plasmids

All RNA oligoribonucleotides were purchased from Genepharma (Shanghai, China). The miRNA duplexes corresponding to mature miR-101 were designed as described previously (6). The siRNAs targeting human *c-Jun* (GenBank accession no. NM_002228), *c-Fos* (NM_005252) and *ERK2* (NM_002745) were designed by the online tool, siDESIGN (Dharmacon, IL, USA). The miR-101 inhibitor (anti-miR-101) with a sequence complementary to the mature miR-101 and its control (anti-NC) were 2′-*O*-methyl-modified RNA oligoribonucleotides.

The *c-Jun* and *c-Fos* expression plasmids, pcDNA-Jun and pcDNA-Fos, were generous gifts from Dr Yosef Shaul (Weizmann Institute of Science, Rehovot, Israel). For construction of lentiviral vector expressing human *c-Jun* and *c-Fos*, the corresponding coding sequences were subcloned into the pCDH-CMV-MCS-EF1-copGFP vector (System Biosciences, CA, USA) and designated as pCDH-c-Jun and pCDH-c-Fos, respectively. For lentivirus production, HEK293T cells were co-transfected with the lentivirus expression vector, pMD2.G and psPAX2 (Addgene) using Lipofectamine 2000, followed by replacement with fresh medium 10 h post-transfection and the supernatant was harvested 48 h after transfection. The lentiviral supernatant was centrifuged (1000 g for 10 min) to remove cellular debris and the aliquots containing the infectious lentiviruses were stored at −80°C until use.

The bioinformatic analysis predicted one conservative miR-101 binding site in ERK2 3′-UTR. A 488-bp wild-type 3′-UTR fragment of human ERK2 mRNA that contained putative binding site for miR-101 was polymerase chain reaction (PCR) amplified from genomic DNA and inserted into XhoI and NotI sites downstream of the stop codon of *Renilla* luciferase gene in psiCHECK-2 vector (Promega, Madison, WI, USA). The mutant 3′-UTR, which contained the mutated sequence in the complementary site for the seed region of miR-101, was generated using fusion PCR based on the construct with wild-type 3′-UTR.

For the *miR-101-2* enhancer reporters, the potential enhancer regions were PCR amplified from genomic DNA, then inserted into XhoI and KpnI sites upstream of the firefly luciferase gene in the pGL3-promoter vector (Promega, Madison, WI, USA). The various deletion constructs that removed potential AP-1 binding sites were generated by fusion PCR based on the constructs with wild-type or single mutant enhancer sequence as depicted in Figure 2A.

**Figure 1.**
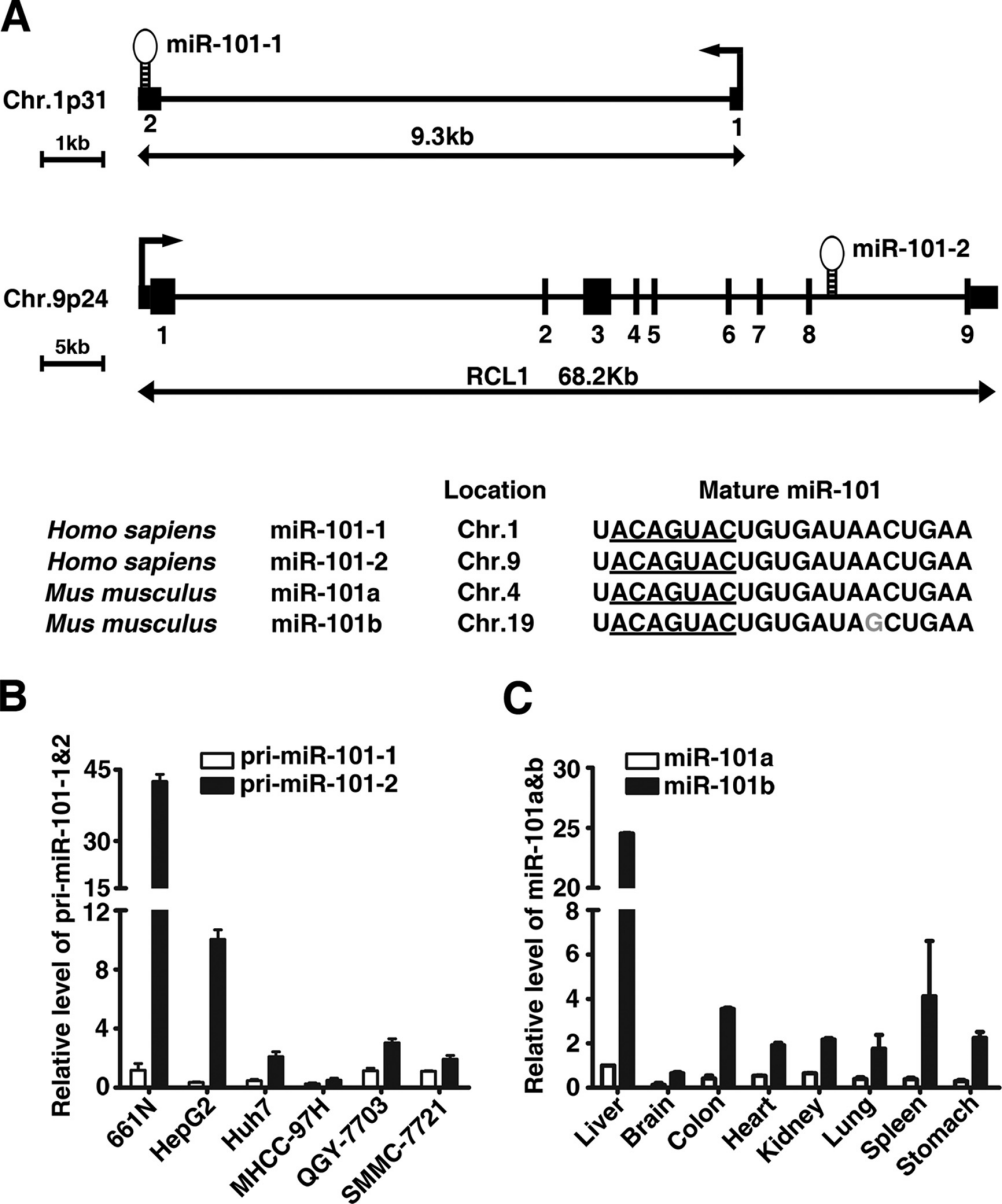
MiR-101 is mainly transcribed from the human *miR-101-2* and mouse *miR-101b* loci. (**A**) Structure and sequence of human miR-101 gene. Sequence data were obtained from the UCSC genome browser database (hg18) and miRBase (release 20). The transcription start site and exon borders of the *miR-101-1* gene are taken from published data (24). Seed region of miR-101 was underlined. The nucleotide that is different in mouse *miR-101b* is indicated in gray color. (**B**) Expression of the primary transcripts of *miR-101-1* and *miR-101-2* genes in normal human liver and hepatoma cell lines. The level of pri-miR-101-1 in the normal human liver (661N) was set to 1. (**C**) Expression of mature miR-101a and miR-101b in various mouse tissues. Expression of miR-101a in the liver was set to 1.

**Figure 2.**
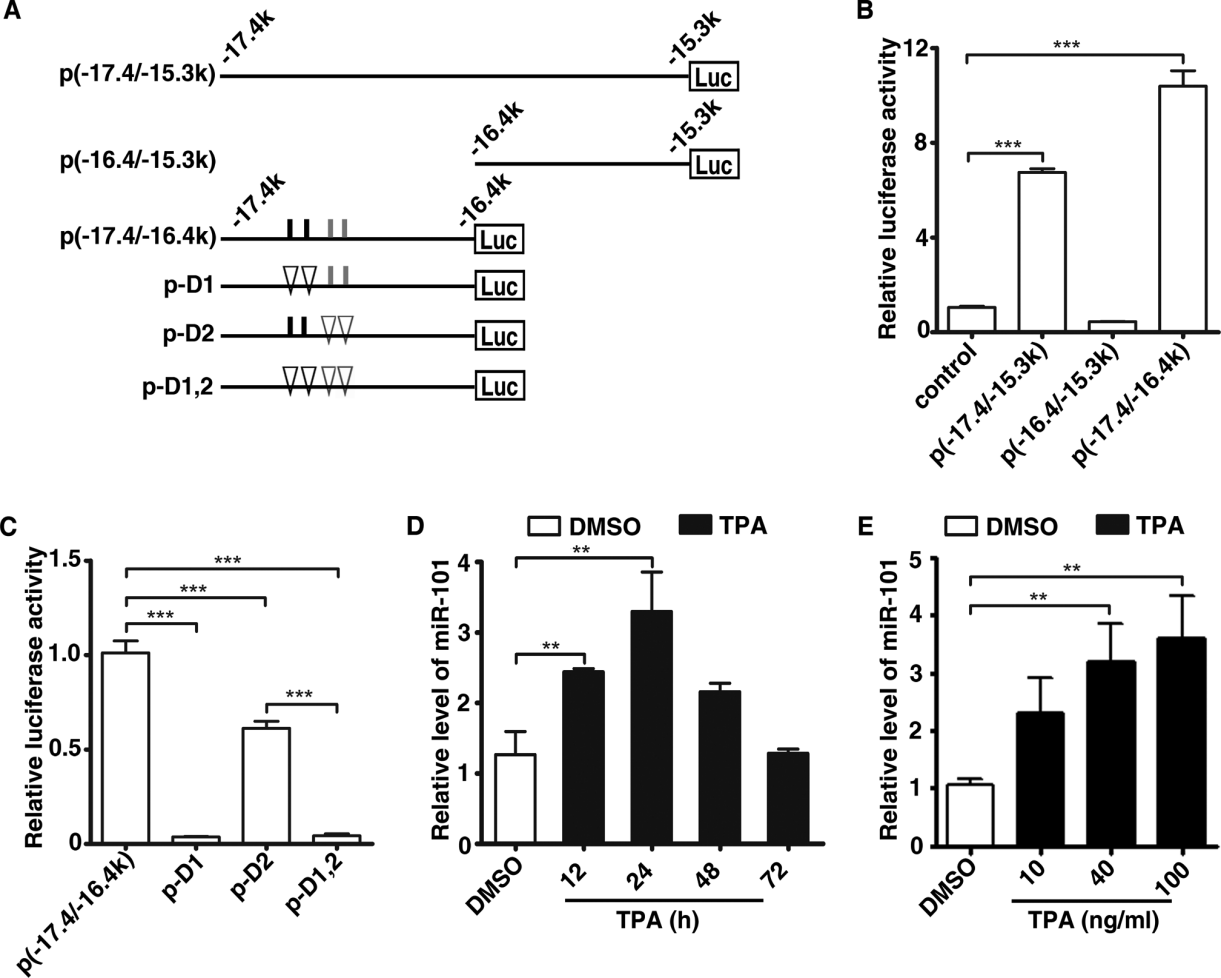
AP-1 is associated with transcription and enhancer activity of miR-101. (**A**) Schematic diagram for luciferase reporter constructs that carry different genomic fragments upstream of miR-101-2 locus. The indicated genomic fragments were cloned into pGL3-promoter plasmid. Putative classical AP-1 binding sites (5′-TGACTCA-3′) are depicted as short black vertical lines, whereas non-classical ones as short gray vertical lines. Deletion of AP-1 binding site is depicted as a triangle. The name of each vector is marked on the left. (**B**) Activity of luciferase reporters containing the indicated genomic regions upstream of miR-101-2. (**C**) Luciferase activity of the various deleted versions of p(−17.4/−16.4 k). In (B) and (C), HepG2 cells were co-transfected with pRL-CMV and the indicated constructs for 48 h, and then subjected to luciferase activity assay. Luciferase activity of one pGL3-promoter or p(−17.4/−16.4k) transfectant was set to 1. ***P < 0.001. (**D**) The miR-101 levels at different time points after TPA exposure. (**E**) The miR-101 levels after treated with different dose of TPA. In (D) and (E), total RNAs were prepared from cells that had been treated with different dose (40 ng/ml in (D)) of TPA for the indicated time periods (24 h in (E)), and then subjected for qPCR. U6 was used as internal control. **P < 0.01.

All wild-type and mutant constructs were verified by sequencing. All RNA oligoribonucleotides and the primers are listed in Supplementary Table S1 and S2, respectively.

### Chromatin immunoprecipitation assay

Briefly, HepG2 cells (5 × 10^6^ each) were treated with TPA (40 ng/ml) for 4 h before cross-linking by formaldehyde. The chromatin complexes were immunoprecipitated using c-Jun or/and c-Fos antibody (Santa Cruz Biotechnology) or normal immunoglobulinG (IgG; as a negative control), then collected by incubation with Dynabeads Protein G (Invitrogen). The DNA–protein cross-link was reversed by heating, and DNA was purified from the eluted solution and subjected to PCR using primers covering the AP-1 binding region. In all experiments, the promoter region of MMP1 including AP-1 binding site was used as positive control (17) and genomic region in the upstream of *miR-101-2* lacking AP-1 binding sites was used as negative control. The sequence of the primers is listed in Supplementary Table S2.

### Electrophoretic mobility shift assay

Electrophoretic mobility shift assay (EMSA) was conducted using a LightShift Chemiluminescent EMSA kit (Thermo Scientific,Pierce, Rockford, IL, USA). The probes, corresponding to the predicted AP-1 binding sequences on the *miR-101-2* enhancer (Supplementary Table S2), were 3′-end-labeled using a Biotin 3′ End DNA Labeling Kit (Thermo Scientific, Pierce, Rockford, IL, USA), then incubated with nuclear extract from TPA-treated HepG2 cells. For competition assay, nuclear extract was preincubated with 400-fold molar excess of unlabeled oligonucleotides prior to adding labeled probe. For antibody-supershift assay, nuclear extract was preincubated with c-Jun or c-Fos antibody or normal IgG before adding to the binding reaction.

### Luciferase assay

For the *miR-101-2* enhancer reporter assays, HepG2 cells in a 48-well plate were cotransfected with 50 ng of the wild-type or mutant reporter plasmids with indicated genomic region, 2 ng pRL-CMV (expressing *Renilla* luciferase), and 50 ng of either GAB (vector control) or mixture with equal amount of pcDNA-Jun and pcDNA-Fos (AP-1) into HepG2 cells. Lysates were collected 48 h post-transfection. Firefly and *Renilla* luciferase activities were measured using the Dual-Luciferase Reporter System (Promega, Madison, WI, USA) according to the manufacturer's instructions. The activity of firefly luciferase in each sample was normalized to that of *Renilla* luciferase. Analysis of the effect of silencing AP-1 on the enhancer activity was performed essentially as described above, except that siRNA targeting *c-Jun* or *c-Fos* was used instead of GAB vector control.

For miR-101 targeted 3′-UTR assays, HepG2 cells in a 48-well plate were co-transfected with 50 ng of the indicated psiCHECK-2 wild-type or mutant reporter plasmids and 50 nM of either miR-101 or control RNA (NC) duplex. After 48 h, the cell lysates were subjected to luciferase activity assays. *Renilla* luciferase activity of each sample was normalized by firefly luciferase activity.

For the AP-1 reporter assays, 50 nM of miR-101, siERK2 or NC were reversely transfected into HepG2 cells for 24 h, then 50 ng of the pAP-1-Luc reporter plasmids (Stratagene, La Jolla, CA, USA) harboring seven copies of AP-1 *cis*-element on its promoter were co-transfected with 15 ng of pRL-PGK (expressing *Renilla* luciferase). Cells were incubated with TPA for 4 h before luciferase activity assays. The activity of firefly luciferase in each sample was normalized to that of *Renilla* luciferase.

### Analysis of gene expression

Real-time quantitative RT-PCR (qPCR) was performed to evaluate gene expression level. The levels of primary miR-101 (pri-miR-101), AP-1 family members and target genes of AP-1, including *MMP1*,*MMP3*, *MMP9*, *CD44*, *uPA*, *uPAR*, *CCND1* and*IL-8*, were quantified by Thunderbird SYBR qPCR Mix (QPS-201, TOYOBO, Japan) and normalized to β-actin expression. Detection of mature miR-101 level was performed on a LightCycler 480 (Roche Diagnostics, Germany) using a TaqMan MicroRNA Assay kit (Applied Biosystems, Foster City, CA, USA). All reactions were run in triplicate. The cycle threshold (*C*_t_) values should not differ by more than 0.5 among triplicates. The mature miR-101 level was normalized to the level of U6 to yield a 2^−△△Ct^ value. Sequences for primers are listed in Supplementary Table S2.

### Western blot analysis

Briefly, total cell lysates were separated by sodium dodecyl sulfate (SDS)-polyacrylamide gels, transferred to poly vinylidene fluoride (PVDF) membranes (Millipore, Billerica, MA, USA) and incubated sequentially with primary and secondary antibodies. Immunoreactive signals were developed with ECL kit (Thermo scientific, Waltham, MA, USA). The antibodies used for immunoblotting were as follows: rabbit monoclonal antibodies (Ab) against c-Fos (9F6), c-Jun (60A8), ERK1/2 (137F5), phospho-ERK1/2 (D13.14.4E), p38 (D13E1), phospho-p38 (D3F9), JNK (56G8) and phospho-JNK (81E11) were from Cell Signaling Technology (Beverly, MA, USA); rabbit polyclonal antibody against phospho-c-Fos (Ser374) (bs-12911R) was from Bioss (Beijing, China); mouse monoclonal Ab against β-actin (BM0627) was from Boster (Wuhan, China).

### Migration and invasion assay

Migration assays were analyzed in a 24-well Boyden chamber with an 8-μm pore size polycarbonate membrane (Corning Glass Works, Corning, NY, USA). In brief, cells were suspended in 100 μl serum-free DMEM and added to the upper chamber, whereas the lower compartment was filled with 600 μl DMEM containing 10% FBS. After incubation at 37°C for the indicated time, cells were fixed and stained with 0.1% crystal violet. The cells on the top layer were removed with a cotton swab. All the migrated tumor cells on the lower surface of the membrane were counted under a microscope. Invasion assay was done by the same procedure, except that the membrane was coated with Matrigel (3432-005-01, R&D Systems, MN, USA) before adding cells to the upper chamber.

### Detection of MMP9 activity by gelatin zymography

The conditioned media from treated cells were collected, centrifuged at 12,000 g for 8 min to remove cell debris and then stored in aliquots at −80°C. To detect the gelatinolytic activity, aliquots of medium were applied to SDS-polyacrylamide gel containing 1 mg/ml gelatin. Cells were counted to correct the loading. After electrophoresis, the gel was soaked in 2.5% Triton X-100 for 1 h at room temperature to remove SDS and then incubated at 37°C overnight in the development buffer. The gel was stained with 0.5% Coomassie brilliant blue R250, followed by destaining and scanning.

### Statistical analysis

Data were presented as the mean ± standard error of the mean from at least three independent experiments. The Student's *t*-test was applied to compare the differences between two groups. When more than two groups were compared, one-way analysis of variance (ANOVA) was applied. All statistical tests were two-sided. A *P* < 0.05 was considered statistically significant.

## RESULTS

### miR-101 is mainly transcribed from the human *miR-101-2* and mouse *miR-101b* loci

The mature miR-101 is processed from two primary miR-101 transcripts in human: pri-miR-101-1 encoded on chromosome 1 and pri-miR-101-2 encoded on chromosome 9 (Figure 1A). To determine the relative abundance of different miR-101 primary transcripts, the expression levels of pri-miR-101-1 and pri-miR-101-2 were analyzed in normal human liver tissue (661N) and five human hepatoma cell lines (HepG2, Huh7, MHCC-97H, QGY-7703 and SMMC-7721). We found that in all samples, the level of pri-miR-101-2 was significantly higher than that of pri-miR-101-1 (Figure 1B). Furthermore, compared with the normal liver, all the hepatoma cell lines showed dramatically decreased pri-miR-101-2 expression (Figure 1B). These data suggest that pri-miR-101-2 is the main precursor of miR-101 in human liver cancer cell lines and normal liver tissue.

In some instances, primary miRNA transcripts may be present only transiently before processing, therefore the primary miRNA level may not represent the transcription level and not correlate to the level of mature miRNA precisely. To exclude this possibility, we checked the expression levels of mature miR-101a and miR-101b in different mouse tissues as they have a nucleotide difference that can be distinguished by the TaqMan MicroRNA Assay Kit. Consistent with the above finding in human cells, the expression of miR-101b, the mouse counterpart of human miR-101-2, is much higher than that of miR-101a in all the mouse tissues examined (Figure 1C), with the highest level of both miR-101a and miR-101b in the liver.

These results suggest that miR-101 is mainly transcribed from the *miR-101-2* locus in human and the*miR-101b* in mouse.

### miR-101 transcription is directly activated by AP-1

To gain further insight into the regulation of pri-miR-101-2 transcription, we first analyzed the ENCODE chromatin immunoprecipitation (ChIP)-sequencing data and found that H3K4me1, H3K4me3 and H3K27Ac, three modifications associated with active enhancers, were extensively enriched in the 17.4 to 15.3 kb region upstream of miR-101-2 precursor (denoted as −17.4 to −15.3 k, with the first base of pre-miR-101-2 assigned as 1, chr9: 4840297). Moreover, three clusters of DNase I hypersensitive sites were included in this region (Supplementary Figure S1), implying there exists potential enhancer within the −17.4 to −15.3 k region. To examine this hypothesis, DNA fragments carrying the −17.4 to −15.3 k (chr9: 4822890–4824940) and its 5′- or 3′-end deletion mutant which contained −17.4 to −16.4 k (chr9: 4822890–4823854) or −16.4 to −15.3 k (chr9: 4823923–4824940), respectively, were cloned upstream of the luciferase gene in the pGL3-promoter reporter vector (Figure 2A). When transfected into HepG2 cells, the luciferase activity of both p(−17.4/−15.3k) and p(−17.4/−16.4k) was greatly elevated, compared to that of the control (Figure 2B). In contrast, the p(−16.4/−15.3k) displayed no enhanced activity, suggesting that the region between −17.4 and −16.4 k contains the critical regulatory element(s) for gene transcription.

We then explored the transcription factors that regulated the −17.4 to −16.4 k region. A prediction of transcription factors identified AP-1 as the one that had the most abundant binding sites in the −17.4 to −16.4 k region (Figure 2A and Supplementary Figure S1). Deletion of the two canonical AP-1 binding sites resulted in almost complete abrogation of the p(−17.4/−16.4 k) activity, and removal of the non-classical binding sites also reduced the activity of p(−17.4/−16.4 k), although to a lesser extent (Figure 2C). These data suggest that AP-1 may regulate the transcription of *miR-101-2* through the −17.4/−16.4k enhancer region.

As AP-1 is an inducible transcription factor complex that can be activated by TPA (18), we therefore treated HepG2 and MHCC-97H cells with TPA to examine whether AP-1 regulated miR-101 expression. As expected, the expression of miR-101 increased at 12 h and peaked at 24 h after TPA treatment (Figure 2D and Supplementary Figure S2A) in both cell lines. Moreover, TPA induced miR-101 expression in a dose-dependent manner (Figure 2E and Supplementary Figure S2B). To explore which AP-1 component involved in TPA-induced miR-101 expression, the expression of the Jun, Fos and ATF family members was determined in TPA-treated HepG2 cells. We found that the mRNA of *c-Jun*, *JunB* and *c-Fos* accumulated in a dose-dependent manner as early as 0.5 h after TPA treatment, and increased further at 1-h time point (Supplementary Figure S3). Accordingly, western blot also showed that c-Fos and c-Jun protein were significantly induced 2 h after TPA treatment, and then decreased gradually (Supplementary Figure S4A and B). The other AP-1 proteins remained largely unchanged or showed very low expression levels except that *ATF2* showed a little downregulation. Since *c-Jun* and *JunB* have similar functions (19) and the former showed relatively higher expression level, we therefore focused on*c-Jun* and *c-Fos* for further study.

**Figure 3.**
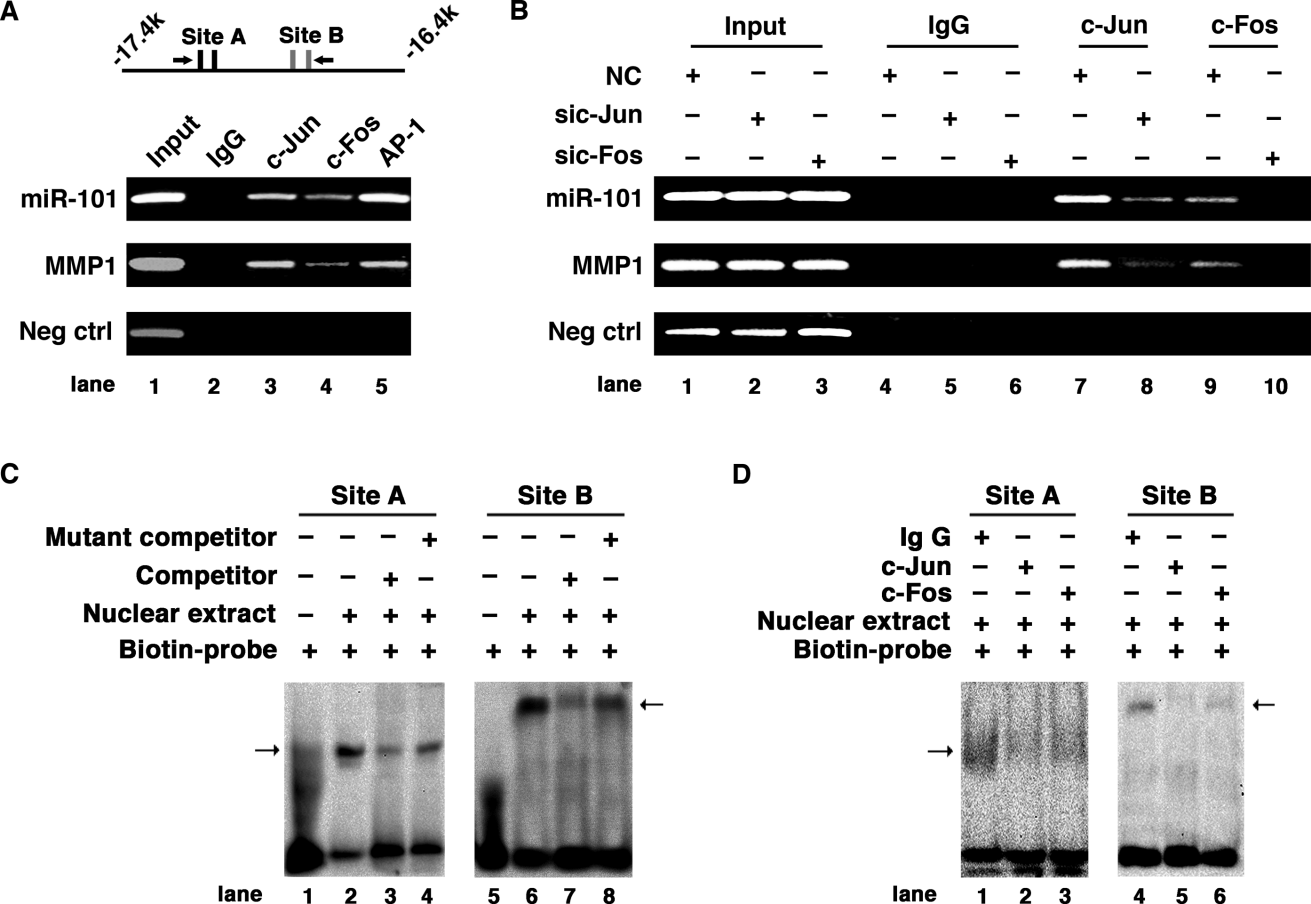
Direct interaction of AP-1 with the enhancer region of *miR-101-2* gene *in vivo* and *in vitro.* (**A**) AP-1 interacts with the enhancer region of *miR-101-2* gene *in vivo.* A scheme of the amplicons is shown at the top. AP-1 binding sites are depicted as short vertical lines and the arrows show the primer set used. (**B**) Knockdown of *c-Jun* or *c-Fos* abrogated the interaction of AP-1 with the enhancer region of *miR-101-2*. In (A) and (B), the promoter region of *MMP1* including AP-1 biding site was used as positive control, and genomic region in the upstream of *miR-101-2* lacking AP-1 binding sites was used as negative control. (**C**) EMSA verified the interaction of nuclear proteins with Site A (canonical AP-1 binding sites) and Site B (non-canonical AP-1 binding sites) sequences of the *miR-101-2* enhancer. (**D**) Antibody-supershift assay identified c-Jun and c-Fos as potential nuclear proteins interacting with Site A and B sequences. In (C) and (D), the arrow indicates the DNA–protein complexes.

To examine the binding of c-Jun and c-Fos to the enhancer of *miR-101-2*, ChIP assays were performed in TPA-treated HepG2 cells. A specific band of the expected size was amplified from either the individual or combination of c-Jun and c-Fos antibody-precipitated DNA, using primers that covered the AP-1 binding sites in the −17.4 to −16.4 k enhancer of *miR-101-2* (Figure 3A). Whereas knockdown of either *c-Fos* or *c-Jun* (Supplementary Figure S5A) markedly reduced the antibody-precipitated DNAs (Figure 3B), suggesting the interaction between AP-1 and the miR-101 enhancer *in vivo*. The direct interaction between c-Jun/c-Fos and canonical (Site A)/non-canonical (Site B) AP-1 binding sites was further verified by EMSA and antibody-supershift assays (Figure 3C and D).

**Figure 4.**
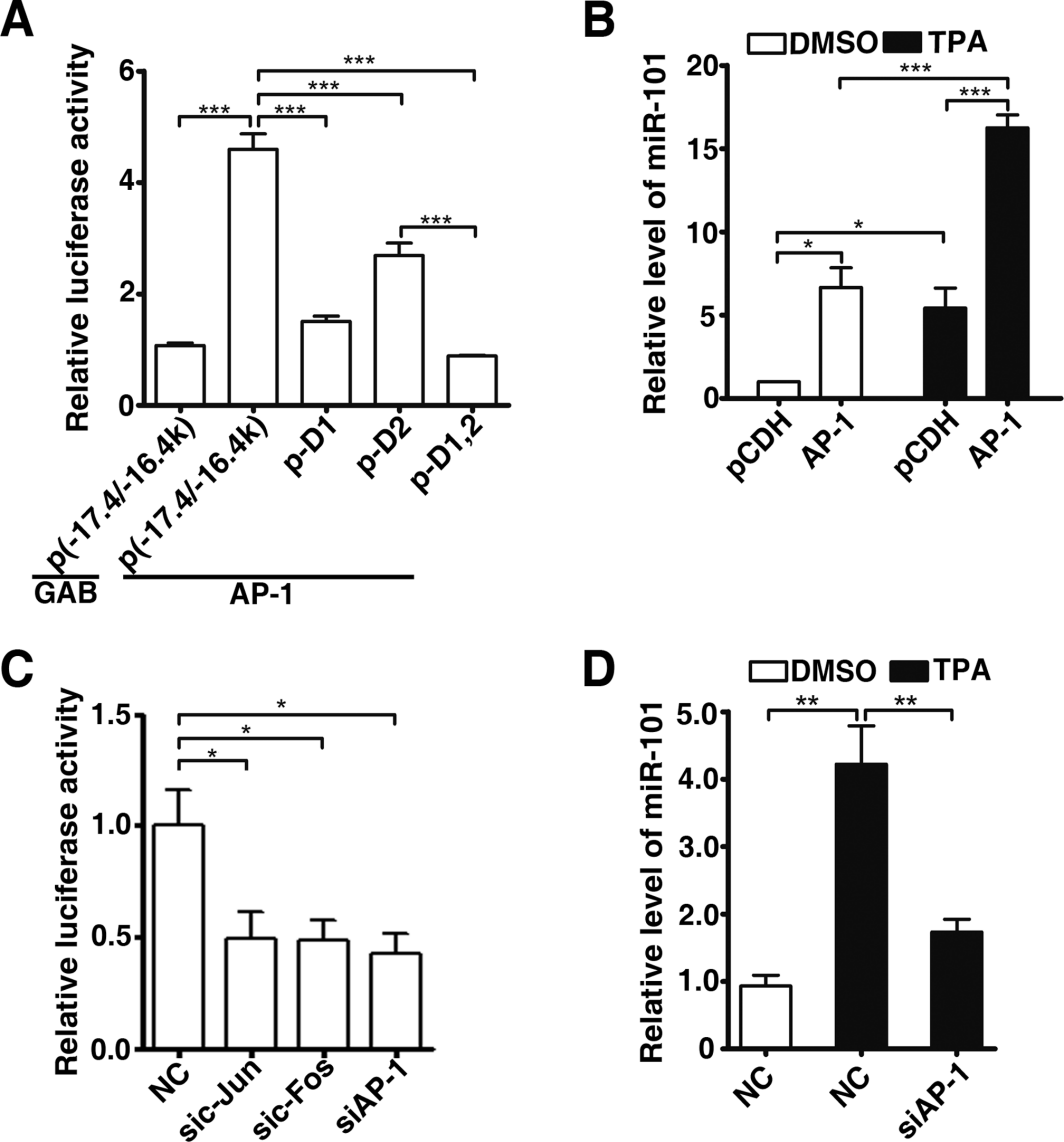
AP-1 regulates miR-101 expression through the enhancer region of *miR-101-2*. (**A**) Effect of AP-1 overexpression on the luciferase activity of the various deleted versions of p(−17.4/−16.4k). HepG2 cells were co-transfected with pRL-CMV and the indicated constructs for 48 h, and then subjected to luciferase activity assay. Luciferase activity of one p(−17.4/−16.4k) transfectant was set to 1. ***P < 0.001. (**B**) Ectopic expression of AP-1 increased miR-101 expression. HepG2 cells were infected with equal titers of lentivirus containing pCDH or mixture of pCDH-c-Jun and pCDH-c-Fos (indicated as AP-1), followed by treatment with DMSO or 40 ng/ml TPA for 48 h. The vector controls (pCDH) treated with DMSO were used for normalization. *P < 0.05, ***P < 0.001. (**C**) Effects of knockdown AP-1 on the luciferase activity of p(−17.4/−16.4k). HepG2 cells were cotransfected with the indicated siRNA, p(−17.4/−16.4k) and pRL-CMV, luciferase activity was measured 24 h later. The mixture of equal amount of sic-Jun and sic-Fos was indicated as siAP-1. NC is negative control *P < 0.05. (**D**) Knockdown of *c-Jun* and *c-Fos* abrogated the TPA-induced miR-101 expression. HepG2 cells transfected with negative control (NC) or mixture of equal amount of sic-Jun and sic-Fos (indicated as siAP-1) were treated with or without 40 ng/ml TPA for 24 h before qPCR assay. **P < 0.01.

Consistently, overexpression of *c-Jun* and*c-Fos* enhanced the activity of p(−17.4/−16.4k) enhancer reporter, which was severely attenuated when the AP-1 binding sites were deleted (Figure 4A). Remarkably, miR-101 level was increased by ectopic expression of *c-Fos* and *c-Jun*, and was further enhanced by TPA treatment (Figure 4B and Supplementary Figure S5B). On the other hand, knockdown of *c-Jun* or/and *c-Fos* led to a dramatic decrease of the transcription activity of p(−17.4/−16.4k) (Figure 4C) and TPA-induced expression of miR-101 (Figure 4D), suggesting that the expression of miR-101 depends on the activity of AP-1.

Collectively, these results indicate that c-Jun and c-Fos may directly bind to the −17.4 to −16.4 k region and promote the expression of miR-101.

### A novel AP-1/miR-101 regulatory feedback loop is implicated in the migration and invasion of hepatoma cells

**Figure 5.**
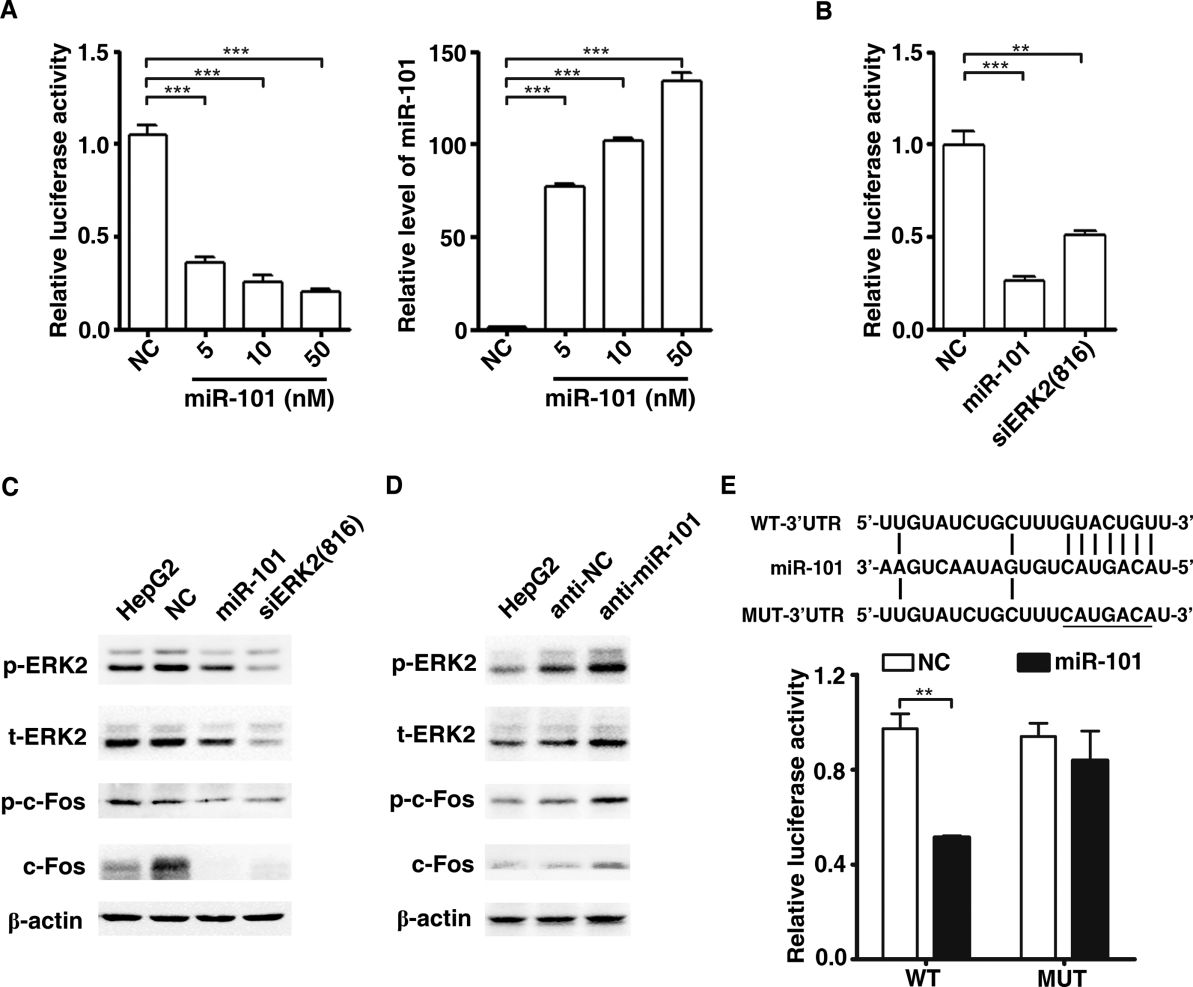
MiR-101 suppresses AP-1 activity by targeting c-Fos and ERK2. (**A**) Overexpression of miR-101 inhibited the transcriptional activity of AP-1 in dose-dependent manner. Left panel: miR-101 suppresses the transcriptional activity of AP-1. Right panel: overexpression effect of miR-101. HepG2 cells were reverse transfected with miR-101 duplex for 24 h, followed by co-transfection with pAP-1-Luc and pRL-PGK plasmids. Luciferase activity was measured 24 h after the transfection of plasmids. All the cells were treated with 40 ng/ml TPA for 4 h before luciferase assay. ***P < 0.001. (**B**) Either ERK2 knockdown or miR-101 overexpression suppressed the transcriptional activity of AP-1. Experiment was performed as described in (A). **P < 0.01, ***P < 0.001. (**C**) miR-101 reduced the levels of cellular c-Fos and ERK2 proteins. Whole cell extracts were prepared from HepG2 cells that had been transfected with the indicated RNA duplexes for 46 h then treated with DMSO or 40 ng/ml TPA for 2 h. β-actin was used as internal control. (**D**) Inhibition of miR-101 increased the expression of c-Fos and ERK2 proteins. HepG2 cells were non-transfected or transfected with the inhibitor of miR-101 (anti-miR-101) or its control (anti-NC) for 44 h, and then treated with TPA for 4 h before protein extraction. (**E**) MiR-101 regulated gene expression by binding to the sequence in the 3′UTR of ERK2. Upper, putative miR-101 binding sequence in the 3′UTR of ERK2 mRNA. Mutation was generated on the potential seed sequence (underlined), as indicated. HepG2 cells were co-transfected with NC or miR-101 duplex, and the luciferase reporter plasmid containing wild-type (WT) or mutant (MUT) ERK2 3′UTR. The normalized luciferase activity of NC transfectant in one experiment was set as relative luciferase activity 1. **P < 0.01.

In a search for the potential targets regulated by miR-101, we found that two key members of the AP-1 signaling pathway, *c-Fos* and *ERK2*, are among the list of predicted targets. To explore the role of miR-101 in AP-1 signaling, HepG2 cells were co-transfected with miR-101 duplex and the luciferase reporter vector harboring seven copies of AP-1 responsive elements (pAP-1-Luc). Overexpression of miR-101 induced a dose-dependent decrease in luciferase activity, an effect mimicked knockdown of *ERK2* (Figure 5A and B). Furthermore, introduction of miR-101 suppressed TPA-induced ERK2 and c-Fos activation (phopho-ERK2 and phopho-c-Fos), as well as total ERK2 and c-Fos proteins (Figure 5C). Consistently, inhibition of endogenous miR-101 by anti-miR-101 increased ERK2 and c-Fos protein levels and prolonged the TPA-induced activation of both ERK2 and c-Fos (Figure 5D). The other MAPKs, including p38 and JNK, were largely unchanged when miR-101 was either overexpressed or depleted (Supplementary Figure S6A and B). As *c-Fos* has been previously reported as a direct target of miR-101 in hepatoma cells (8), we only performed luciferase reporter assays to verify whether miR-101 regulated *ERK2* expression by binding to its 3′-UTR. Transfection of miR-101 significantly suppressed the *Renilla* luciferase activity of the psiCHECK-2 reporter with wild-type but not mutant 3′-UTR of *ERK2* (Figure 5E). These results indicate that miR-101 may suppress AP-1 activity by directly inhibiting the expression of*ERK2* and *c-Fos*.

**Figure 6.**
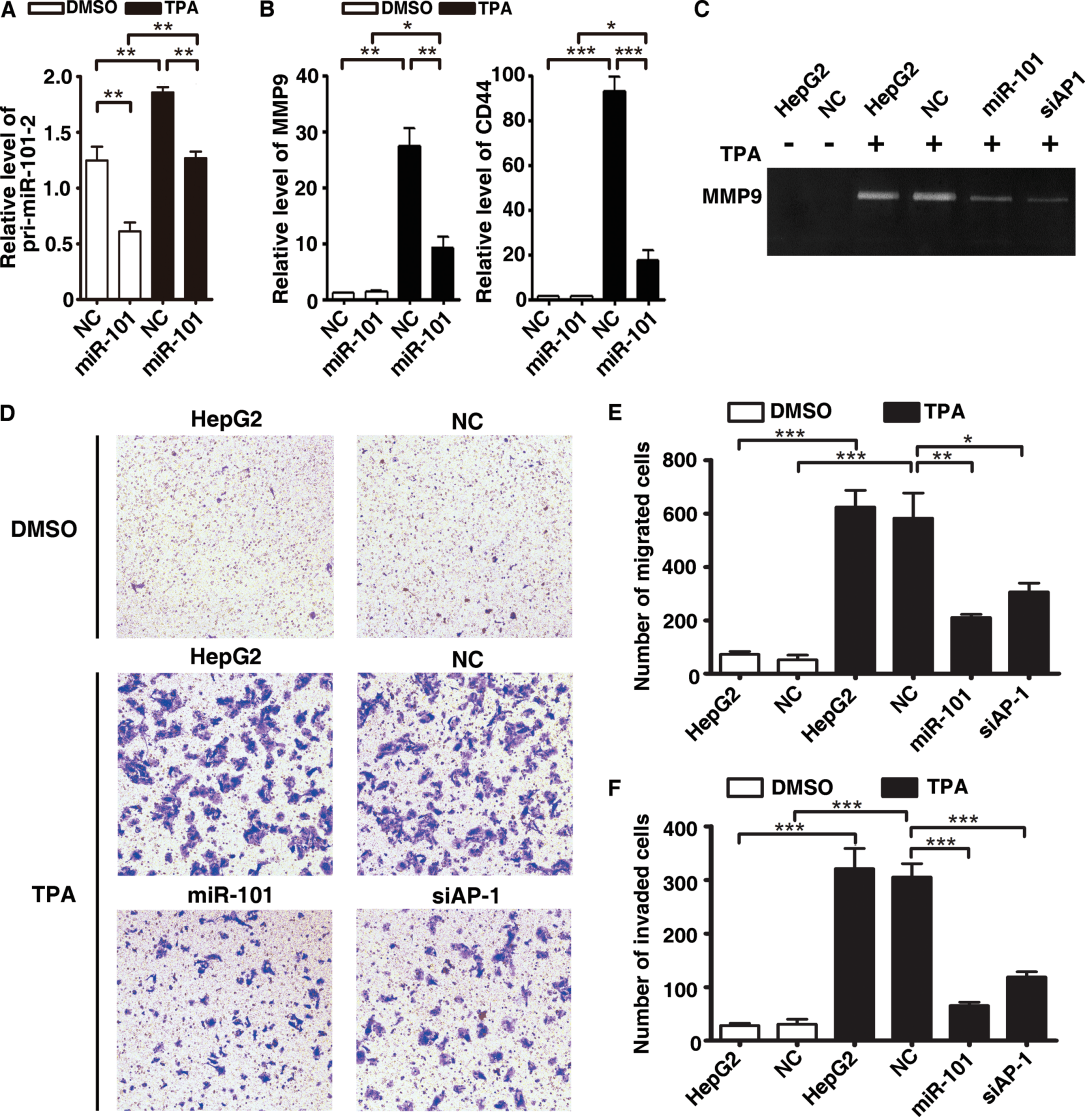
The AP-1/miR-101 regulatory feedback loop regulates the migration and invasion of hepatoma cells. (**A**) Introduction of mature miR-101 efficiently suppressed the transcription of pri-miR-101-2. HepG2 cells transfected with miR-101 mimics were treated with or without 40 ng/ml TPA for 8 h before qPCR assays. **P < 0.01. (**B**) Ectopic expression of miR-101 suppressed TPA-induced expression of MMP9 and CD44. HepG2 cells transfected with NC or miR-101 were treated with DMSO or TPA for 24 h, then subjected for qPCR. The expression level of NC transfectant treated with DMSO was set as 1. *P < 0.05, **P < 0.01 and ***P < 0.001. (**C**) MiR-101 attenuated the TPA-induced MMP9 activity. HepG2 cells without transfection or transfected with RNA duplex for 24 h were treated with or without TPA for 24 h, and then incubated in the serum-free DMEM for 20 h, followed by gelatin zymography assay. (**D**) MiR-101 inhibited the TPA-induced migration of HepG2 cells. (**E**) Quantification of the number of migrated cells as shown in (D). (**F**) MiR-101 repressed the TPA-promoted invasion of HepG2 cells. In (D)–(F), HepG2 cells without transfection or transfected with NC, miR-101 or siRNA duplex were treated with TPA for 24 h, then added to transwell chambers and incubated for 20 h, followed by staining with crystal violet. *P < 0.05, **P < 0.01 and ***P < 0.001.

Our findings that AP-1 pathway and miR-101 were regulated in a reciprocal fashion raised the possibility that AP-1 and miR-101 form a regulatory feedback loop. To verify this hypothesis, we reintroduced the mature miR-101 mimics into HepG2 cells and analyzed the pri-miR-101-2 level. The results showed that introduction of miR-101 efficiently suppressed the TPA-induced transcription of pri-miR-101-2 (Figure 6A). To explore the biological significance of the AP-1/miR-101 regulatory loop, the effect of miR-101 on the expression of several reported AP-1 target genes was firstly examined by qPCR. Among the TPA-induced AP-1 targets, *MMP1*, *MMP3*, *MMP9*, *CD44* and *IL-8* were suppressed by miR-101 (Figure 6B and Supplementary Figure S7). This observation suggests that AP-1/miR-101 may regulate multiple downstream genes and might affect different aspects of cellular function.

**Figure 7.**
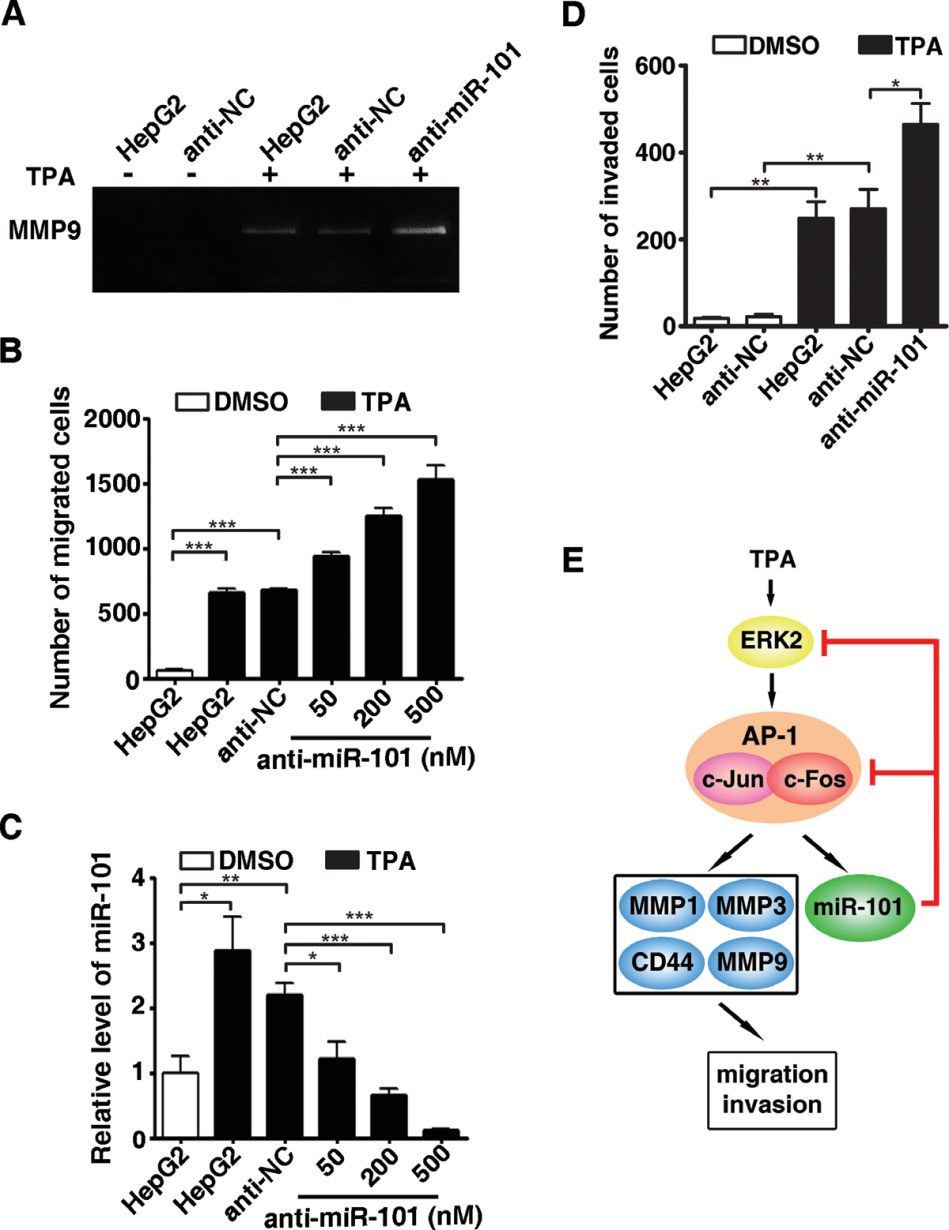
Disruption of the miR-101/AP1 feedback loop promotes the TPA-induced migration and invasion. (**A**) Inhibition of miR-101 increased the TPA-induced MMP9 activity. HepG2 cells without transfection or transfected with anti-miR-101 or anti-NC were treated with or without TPA for 24 h, and then incubated in the serum-free DMEM for 20 h, followed by gelatin zymography assay. (**B**) Antagonism of miR-101 increased the TPA-induced migration in a dose-dependent manner. (**C**) Knockdown effect of anti-miR-101. (**D**) Antagonism of miR-101 increased the TPA-induced invasion. In (B) and (D), HepG2 cells without transfection or transfected with anti-miR-101 or anti-NC were treated with or without TPA for 24 h, and then added to transwell chambers for 20 h, followed by analysis for migration (B) and invasion (D). *P < 0.05, **P < 0.01 and ***P < 0.001. (**E**) Schematic diagram showed the AP-1/miR-101 regulatory feedback loop.

Considering that CD44 and MMPs are critical factors in regulating cell adhesion and Extracellular matrix (ECM)-degrading and gelatin zymography disclosed that both miR-101 and siAP-1 significantly inhibited the TPA-enhanced MMP9 activity (Figure 6C), we therefore evaluated whether miR-101 could inhibit AP-1-mediated migration and invasion. Transwell assay showed that TPA significantly enhanced the migration and invasion of HepG2 cells, which were effectively abrogated by miR-101 and siAP-1 (Figure 6D–F). Consistently, the antagonism of miR-101 increased the MMP9 activity (Figure 7A) and in turn promoted the migration and invasion of TPA-treated HepG2 cells (Figure 7B–D). Taken together, we suggest a novel AP-1/miR-101 regulatory circuitry: AP-1 promotes the transcription of miR-101, whereas the expression of miR-101 reduces the level of ERK2 and c-Fos and thereby attenuates the AP-1 signaling (Figure 7E). These findings indicate that miR-101 may prevent the excessive activation of metastatic signals imposed by ERK2/AP-1 and the AP-1/miR-101 regulatory loop thus plays a vital role in maintaining cellular homeostasis.

## DISCUSSION

In this study, we disclose a novel AP-1/miR-101 regulatory circuitry, that is, AP-1 promotes the transcription of miR-101, whereas the expression of miR-101 reduces the levels of ERK2 and c-Fos and thereby attenuates the AP-1 signaling and in turn abrogates the AP-1-promoted migration and invasion of cancer cells. Our results elucidate the regulation mechanism of miR-101 transcription and suggest an important role of the AP-1/miR-101 feedback loop in preventing the excessive activation of metastatic signals imposed by ERK2/AP-1.

Although the tumor suppressive role of miR-101 has been reported in a wide variety of cancers, how the expression of miR-101 itself is regulated remains elusive. There are two genomic loci encode miR-101 in human, including *miRNA-101-1* on chromosome 1 and *miRNA-101-2* on chromosome 9. *MiR-101-1* is located in intergenic region and *miR-101-2* in the eighth intron of *RCL1* gene. Organization of both *miR-101-1* and *miR-101-2* genes is highly conserved between human and mouse. Based on the results from qPCR analysis in human hepatoma cell lines and mouse tissues, we showed that miR-101 was mainly transcribed from human *miR-101-2* and mouse *miR-101b* gene loci. Furthermore, we identified the enhancer region for the *miR-101-2* gene and classified AP-1 as a transactivator of miR-101 expression rested on the following evidence: Firstly, ectopic expression of AP-1 or stimulation with TPA, an AP-1 activator significantly increased miR-101 level and knockdown of AP-1 abrogated the TPA-induced miR-101 expression. Secondly, the −17.4/−16.4 k region of *miR-101-2* carried an enhancer activity, which was attenuated by deleting the AP-1 binding sites in this region. Thirdly, c-Jun and c-Fos directly interacted with the enhancer region of *miR-101-2* *in vitro* and *in vivo*. Taken together, our study delineates a novel mechanism underlying the transactivation of *miR-101 gene*.

The AP-1 components, *c-Jun* and *c-Fos* are immediate early response genes that can be activated by a variety of external stimuli or cellular stress. In normal cells, the AP-1 signaling is precisely controlled to maintain cellular homeostasis. Both*c-Jun* and *c-Fos* undergo the proteasome-mediated degradation soon after they are activated, thus ensure the proper termination of AP-1 signaling (20). As key post-transcriptional regulators of gene expression, miRNAs have been reported to orchestrate the regulation of cellular signaling networks by acting as critical motifs of feedback circuitry (21–23), which warrant the robustness and stability in the context of dynamic changes in cells. Here we showed that miR-101, a direct target of AP-1, could attenuate the AP-1 signaling by directly inhibiting the expression of ERK2 and c-Fos. Our results suggest that the miR-101 feedback regulation on AP-1 signaling is an alternative mechanism in preventing the prolonged AP-1 activity.

It is worth noting that downregulation of miR-101 is a common event in cancers and has been implicated in the development and progression of different malignancies. Allele loss is one of the main mechanisms of decreased miR-101 expression in prostate tumors, which was also implicated in a subset of other cancers like breast, gastric cancers and glioblastoma (5). In addition, studies in HCCs and bladder cancers have demonstrated that miR-101 is epigenetically repressed by EZH2-mediated histone modification (10,24), which may also lead to the aberrant regulation of AP-1 signaling by miR-101.

It has been shown that c-Jun promotes cell proliferation via transactivating cyclin D1 or suppressing p16^INK4A^ expression (25) and inhibits apoptosis by repressing p53 activation (26). Extensive evidence also suggests an important role of AP-1 in tumor metastasis. Conditional expression of an inducible c-FosER fusion protein in murine breast epithelial cells triggers the epithelial-fibroblastoid cell conversion and enhances invasion *in vitro* (27). In contrast, c-Fos-deficient mice fail to form invasive carcinoma in mouse skin tumorigenesis models (28). Furthermore, several matrix metalloproteases, including *MMP1*, *MMP3* and *MMP9*, have long been verified as the direct targets of AP-1 in a variety of cellular contexts (16). *CD44*, the hyaluronan receptor, has been identified as another target of AP-1 that mediated cell adhesion required for tumor cell migration as well as growth and dissemination of a variety of tumor types (29,30). Recent studies also suggest a potential role of AP-1 in tumor angiogenesis. Oxidative stress can provoke AP-1 activity in HCC cell lines, leading to the induction of *vascular endothelial growth factor* (*VEGF*) and *IL-8*, which are considered as key factors for angiogenesis (31). Likely, it was shown that c-Fos and c-Jun dimer promotes the interleukin-7-induced lymphangiogenesis in lung cancer by upregulating VEGFD expression (32). In agreement with these findings, a number of human cancers exhibit increased expression of *c-Jun* and *c-Fos* (25). Amplification of *c-Jun* is reported as one cause of AP-1 activation in undifferentiated and aggressive human sarcomas (33). The Ras/Raf/MEK/ERK signal transduction pathway is frequently activated by mutations in its components in many types of cancers, which results in its sustained stimulation and a subsequent increase in transcription and stability of AP-1 (34). Our data suggest that frequent downregulation of miR-101 may also account for the abnormal activation of AP-1 signaling in human cancers.

Interestingly, removal of the AP-1 binding sites in p(−17.4/−16.4k) construct led to a less profound decrease of the enhancer activity in the AP-1-overexpressed cells compared with the non-transfected cells. This potential discrepancy may be explained by the following possibilities: (i) in the cells with basic AP-1 activity, the non-classical AP-1 binding sites depend largely on the proximal classical AP-1 binding sites to recruit AP-1, thus removal of classical AP-1 binding sites leads to a dramatic reduction of luciferase activity. Whereas AP-1 overexpression may facilitate the recruitment of AP-1 for the non-classic binding sites even the canonical ones are removed; (ii) the presence of excessive AP-1 may interact with other transcriptional factors that also have binding site in the −17.4/−16.4k region and consequently enhance luciferase activity, thus partially rescue the effect caused by the removal of the AP-1 binding sites.

Taken together, we disclose a novel AP-1/miR-101 regulatory circuit based on *in vitro* and *in vivo* assays. Our results suggest that this circuitry may effectively protect cells from the abnormal transcription of pro-metastatic genes and therefore plays a vital role in maintaining cellular homeostasis.

## SUPPLEMENTARY DATA

Supplementary Data are available at NAR Online.

SUPPLEMENTARY DATA
