# Supplementary material for: A novel AP-1/miR-101 regulatory feedback loop and its implication in the migration and invasion of hepatoma cells
Source: Nucleic Acids Res. 2014 Sep 26;42(19):12041–51. doi: 10.1093/nar/gku872 (PMC4231742; doi:10.1093/nar/gku872)
Supplement: SUPPLEMENTARY DATA [file supp_42_19_12041__index.html]

A novel AP-1/miR-101 regulatory feedback loop and its implication in the migration and invasion of hepatoma cells — A novel AP-1/miR-101 regulatory feedback loop and its implication in the migration and invasion of hepatoma cells — SUPPLEMENTARY DATA 

# A novel AP-1/miR-101 regulatory feedback loop and its implication in the migration and invasion of hepatoma cells

## SUPPLEMENTARY DATA

**Files in this Data Supplement:**

- SUPPLEMENTARY DATA
- SUPPLEMENTARY DATA
