# Supplementary material for: A novel AP-1/miR-101 regulatory feedback loop and its implication in the migration and invasion of hepatoma cells
Source: Nucleic Acids Res. 2014 Sep 26;42(19):12041–51. doi: 10.1093/nar/gku872 (PMC4231742; doi:10.1093/nar/gku872)

Supplementary Figure Legends

Supplementary Figure 1. The sequence features of -17.4/-15.3k region of miR-101-2 gene. The relative positions of exons and introns of RCL1 are indicated as blue filled boxes and solid lines, respectively. Histograms show the profiles of H3K4me1, H3K4me3 and H3K27Ac in cell lines. Clusters of DNase I hypersensitive sites are showed as horizontal filled bars and the predicted AP-1 binding sites are depicted as short vertical lines.

Supplementary Figure 2. AP-1 regulates miR-101 expression in MHCC-97H cells. (A) The miR-101 levels at different time points after TPA exposure. (B) The miR-101 levels after treated with different dose of TPA. In A and B, cells were treated with DMSO or 40 ng/ml TPA for the indicated time periods, and then subjected for qPCR assay. * $P < 0.05$, ** $P < 0.01$.

Supplementary Figure 3. Dose-response of JUN, FOS and ATF family genes expression in HepG2 cells after exposure to TPA. Cells were incubated in the presence of TPA at varying concentrations for 0.5 hour (upper) or 1 hour (lower), and then subjected for qPCR assay. The level of c-Fos in DMSO treated cells in each time point was set to 1. * $P < 0.05$, ** $P < 0.01$, *** $P < 0.001$.

Supplementary Figure 4. Expression patterns of c-Jun and c-Fos proteins in TPA-treated HepG2 (A) and MHCC-97H cells (B). Cells were treated with 40 ng/ml TPA for the indicated time periods. DMSO was used as vehicle control.

Supplementary Figure 5. Knockdown (A) or overexpression (B) effect of c-Jun and c-Fos. In A, HepG2 cells were nontransfected or transfected with the indicated duplex, and then treated with 40 ng/ml TPA for 2 hours before Western blotting assay. In B, HepG2 cells were infected with equal titers of lentivirus containing pCDH or mixture of pCDH-c-Jun and pCDH-c-Fos (indicated as AP-1). Whole cell lysates were subjected to Western blot analysis 72 hours later. β-actin was used as internal control.

Supplementary Figure 6. Effects of miR-101 and miR-101 inhibitors (anti-miR-101) on endogenous p38 and JNK levels in HepG2 cells treated with TPA. (A) Effects of miR-101 on endogenous p38 and JNK levels in TPA treated HepG2 cells. The experiment was the same as illustrated in Figure 5D. (B) Effects of miR-101 inhibitors on endogenous p38 and JNK levels in HepG2 cells treated with TPA. The experiment was the same as illustrated in Figure 5E.

Supplementary Figure 7. Effects of miR-101 on the expression of AP-1 target genes in TPA-treated HepG2 cells. HepG2 cells transfected with NC or miR-101 were treated with DMSO or TPA for 24 hours, then subjected for qPCR. The expression level of NC-transfectant treated with DMSO was set as 1. * $P < 0.05$, ** $P < 0.01$, *** $P < 0.001$.

Supplementary Figure 1

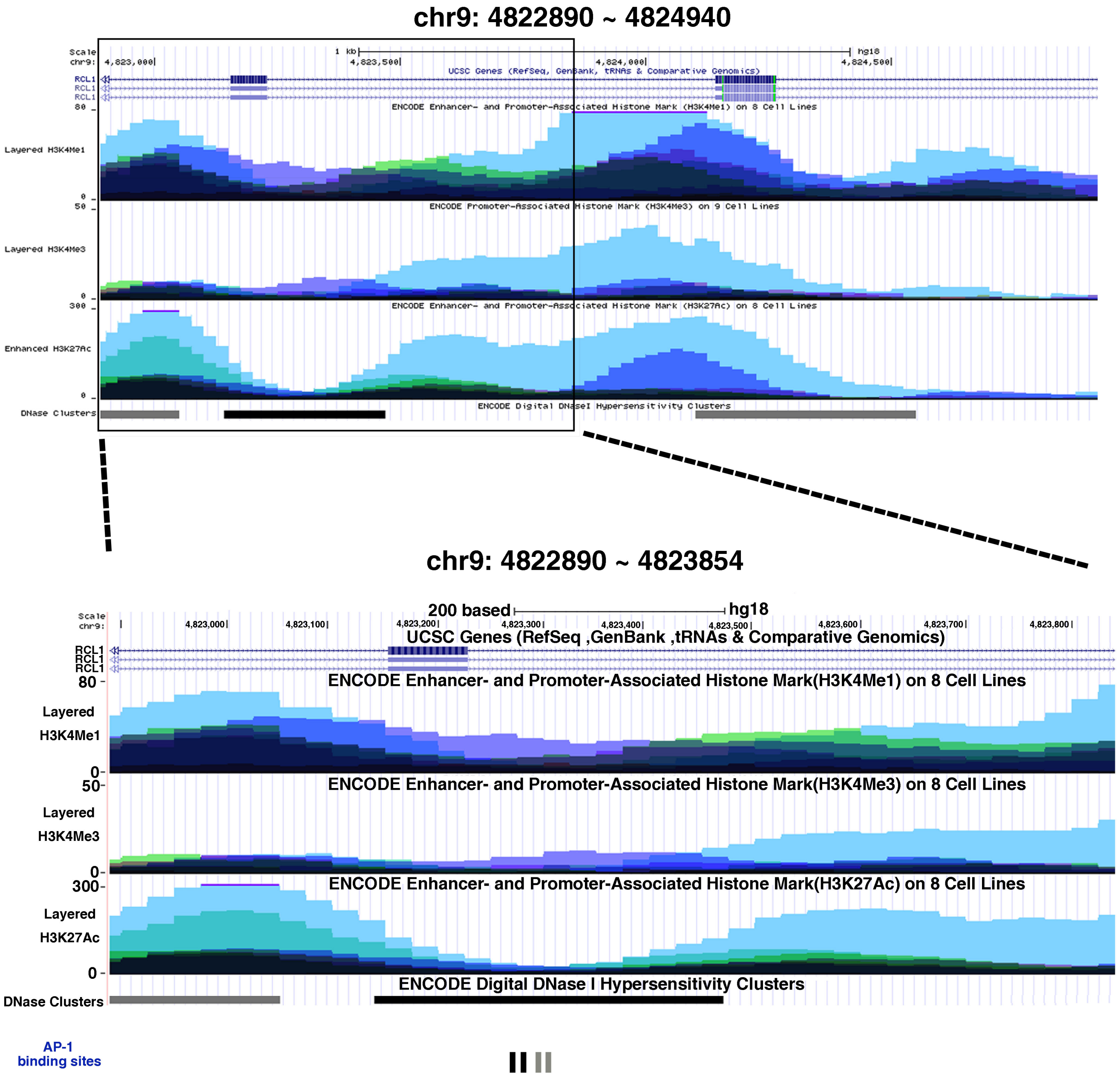

Supplementary Figure 2

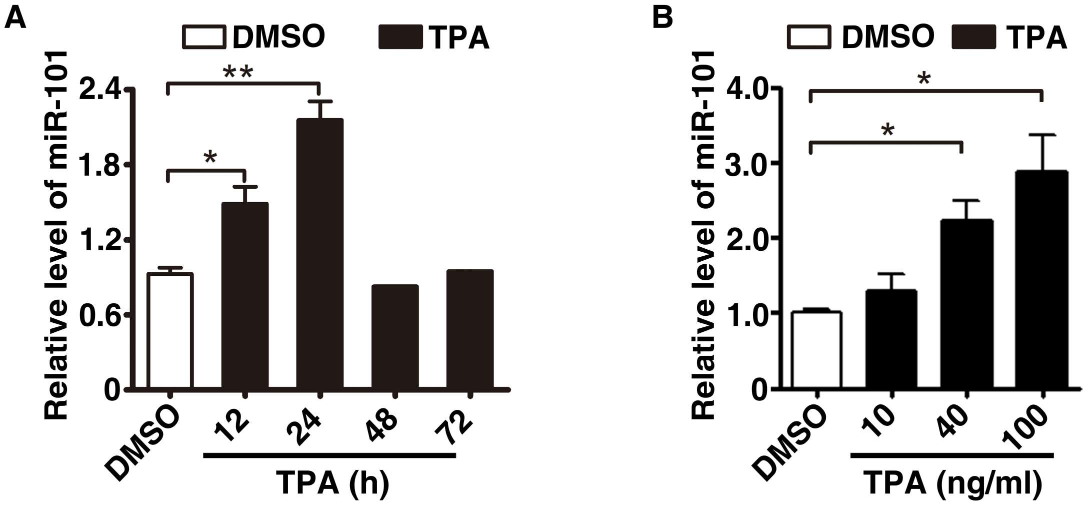

Supplementary Figure 3

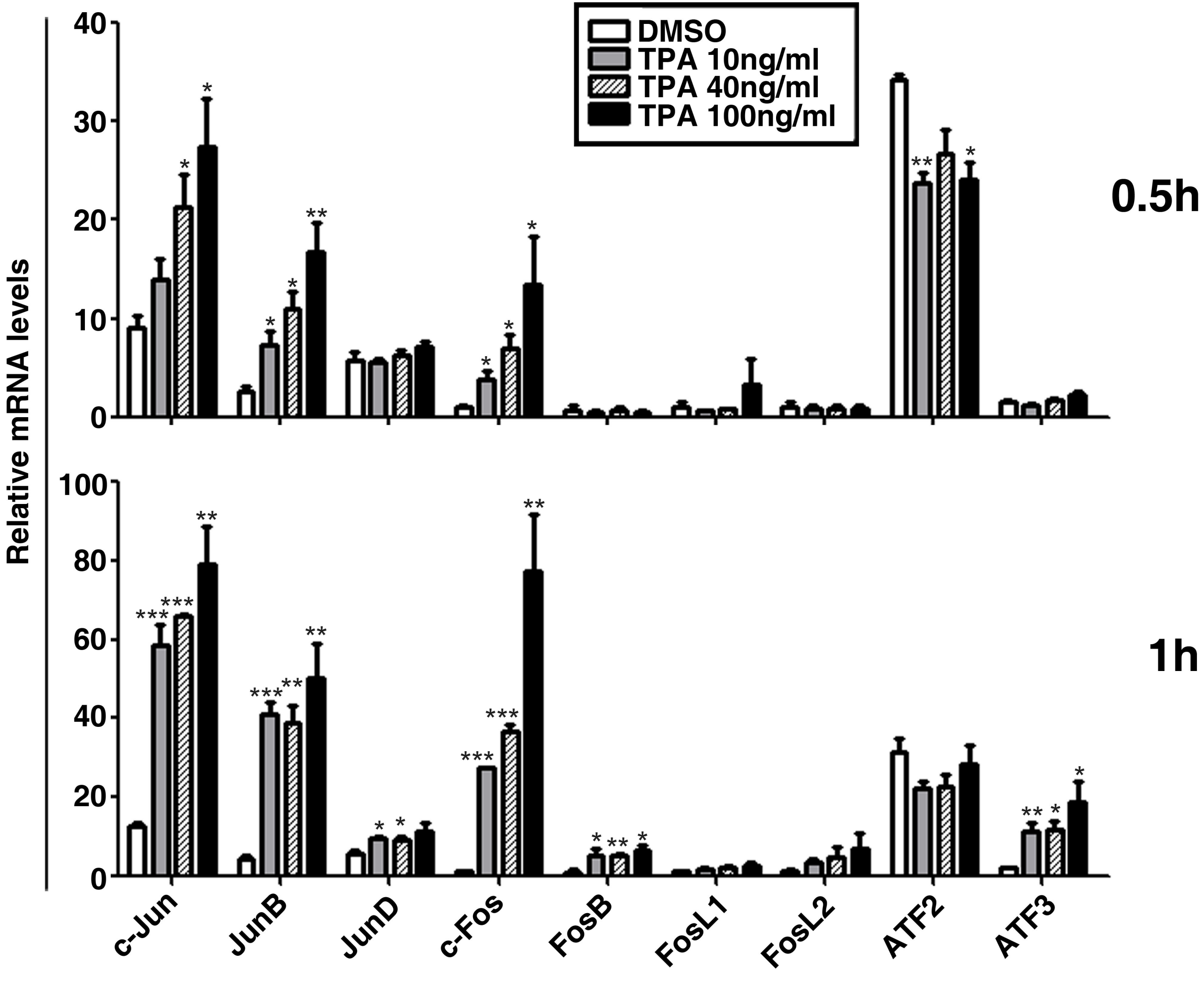

Supplementary Figure 4

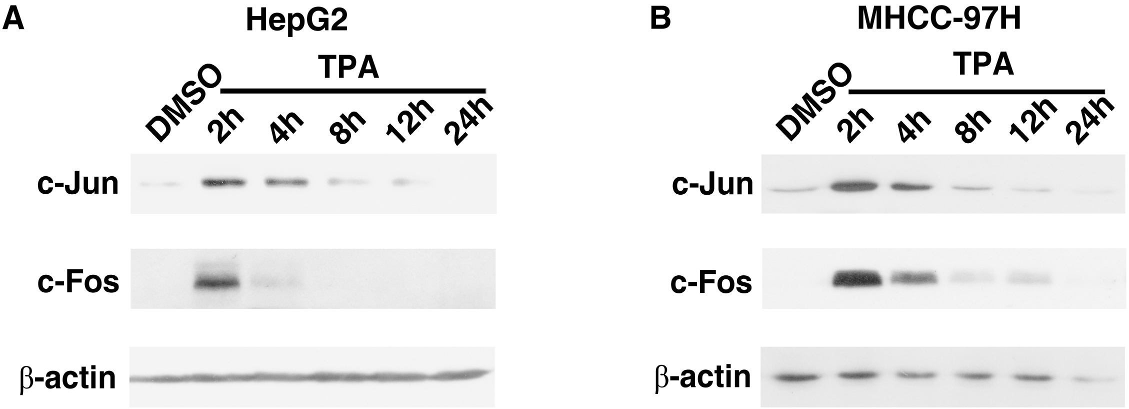

Supplementary Figure 5

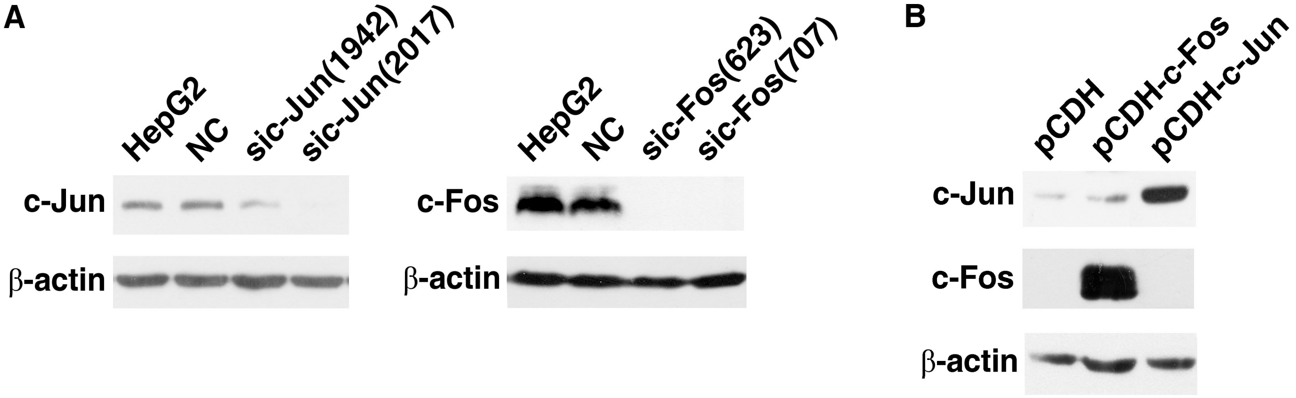

Supplementary Figure 6

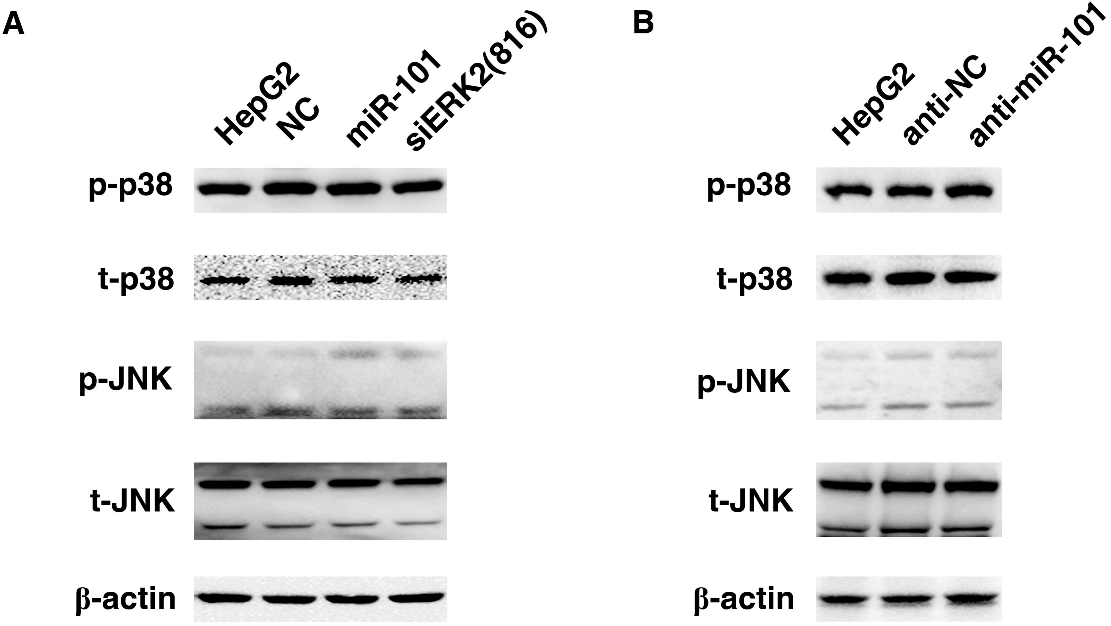

Supplementary Figure 7

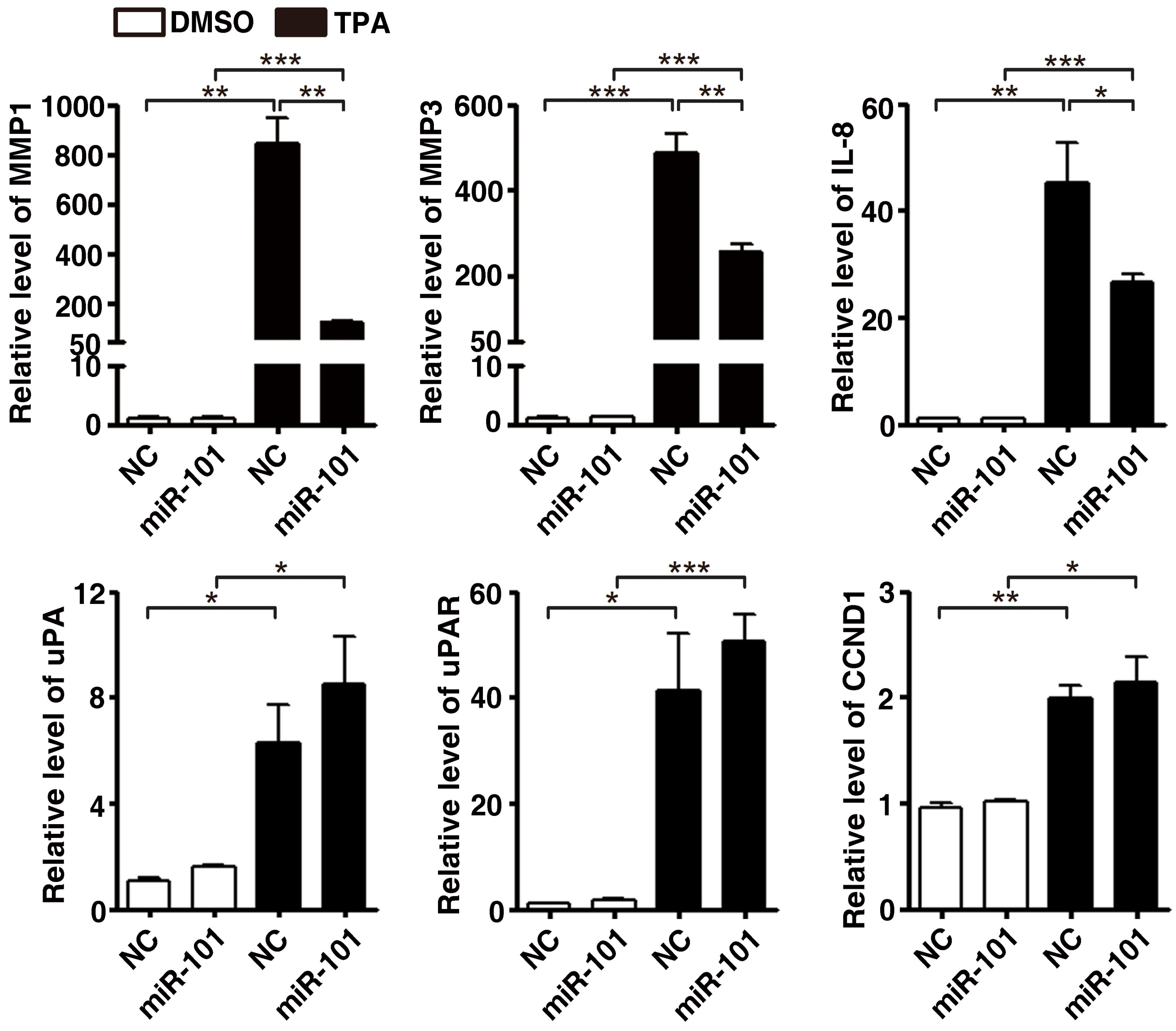

Supplement: SUPPLEMENTARY DATA [file supp_gku872_nar-02333-x-2014-File010.pdf]
