# Supplementary material for: A novel AP-1/miR-101 regulatory feedback loop and its implication in the migration and invasion of hepatoma cells
Source: Nucleic Acids Res. 2014 Sep 26;42(19):12041–51. doi: 10.1093/nar/gku872 (PMC4231742; doi:10.1093/nar/gku872)
Supplement: SUPPLEMENTARY DATA [file supp_gku872_nar-02333-x-2014-File009.docx]

**Supplementary Table 1. Sequences of RNA Oligonucleotides**

| **Name** | **Sense Strand/Sense Primer (5'-3')** | **Antisense Strand/Antisense Primer (5'-3')** |
| --- | --- | --- |
| **RNA oligoribonucleotides** | |  |
| miR-101 | UACAGUACUGUGAUAACUGAA | UCAGUUAUCACAGUACUGUCUU |
| sic-Fos(623) | AGGAGAAUCCGAAGGGAAAdTdT | UUUCCCUUCGGAUUCUCCUdTdT |
| sic-Fos(707) | GAGACAGACCAACUAGAAGdTdT | CUUCUAGUUGGUCUGUCUCdCdG |
| sic-Jun(1942) | UGCUCAGGGAACAGGUGGCdTdT | GCCACCUGUUCCCUGAGCAdTdG |
| sic-Jun(2017) | CGCAGCAGUUGCAAACAUUdTdT | AAUGUUUGCAACUGCUGCGdTdT |
| siERK2(816) | GUACAGGGCUCCAGAAAUUdTdT | AAUUUCUGGAGCCCUGUACdCdA |
| NC | UUGUACUACACAAAAGUACUG | GUACUUUUGUGUAGUACAGUU |
| anti-NC | GUGGAUAUUGUUGCCAUCA |  |
| anti-miR-101 | UUCAGUUAUCACAGUACUGUA |  |

**Supplementary Table 2. Sequences of DNA Oligonucleotides**

| **Name** | **Sense Strand/Sense Primer (5'-3')** | **Antisense Strand/Antisense Primer (5'-3')** |
| --- | --- | --- |
| **Primers for cloning (Restriction enzyme sites were underlined)** | |  |
| p(-17.4/-16.4k) | CACGGTACC CCTCTGATGCACACGATGAC | AGTCTCGAG GTCCATCATCTGAGCCCATT |
| p(-16.4/-15.3k) | GTAGGTACCCACTTGGGTAATCGACTTGG | GATCTCGAGCACAGACCAGGTTCAAATCC |
| ERK2 3'UTR | AGTGAATTCCTCCTGTGGTGCAGATGAGA | AGTTCTAGATCAGGTGCCATAAACATTCAA |
| pCDH-c-Fos | CATGAATTCGCCACCATGATGTTCTCGGGCTTCAAC | ATGGGATCCTCACAGGGCCAGCAGCGTGGGT |
| pCDH-c-Jun | CATGAATTCGCCACCATGACTGCAAAGATGGAAACG | ATGGGATCCTCAAAATGTTTGCAACTGCTGC |
| **Primers for qPCR** | | |
| pri-miR-101-1 | AGCCACCAGAAAGGATGCCGTT | TCTCCCTATGCCCCGGGAGAAGT |
| pri-miR-101-2 | GGCCCATCTGAGGTTGGTGAGC | TAGCCCCACCGCAGTCCTCAA |
| c-Jun | AACAGAGCATGACCCTGAAC | GACTGGATTATCAGGCGCTC |
| JunB | CCACGACGACTCATACACAG | TGACCAGAAAAGTAGCTGCC |
| JunD | ACGAGCTCACAGTTCCTCTA | GCTTGTGTAAATCCTCCAGGG |
| c-Fos | CAGACTACGAGGCGTCATCC | CGTGGGAATGAAGTTGGCAC |
| FosB | CCCTCTGCCGAGTCTCAATA | GAAGAGATGAGGGTGGGTTG |
| FosL1 | ATCAACACCATGAGTGGCAG | CGGGCTGATCTGTTCACAAG |
| FosL2 | GATCACCTCCATGTCCAACC | ACGCTTCTCCTCCTCTTCAG |
| ATF2 | TTCTGCCAGGCAATACAAGG | CACTGTCATTACGTGCTGGA |
| ATF3 | TCCATCACAAAAGCCGAGGT | CTTGTTTCGGCACTTTGCAG |
| MMP1 | ATGAAGATGAAAGGTGGACC | GTACATCAAAGCCCCGATAT |
| MMP3 | TTACCCTTTTGATGGACCTG | AATTGGTCCCTGTTGTATCC |
| MMP9 | ACGCAGACATCGTCATCCAGT | GGACCACAACTCGTCATCGTC |
| CD44 | cttcaacccaatctcacacc | tcttccaccaaacctgtgtt |
| uPA | TCACCACCAAAATGCTGTGT | AGGCCATTCTCTTCCTTGGT |
| uPAR | TGAAGAACAGTGCCTGGATG | TGTTGCAGCATTTCAGGAAG |

**Supplementary Table 2. Sequences of DNA Oligonucleotides (Continued)**

| **Name** | **Sense Strand/Sense Primer (5'-3')** | **Antisense Strand/Antisense Primer (5'-3')** |
| --- | --- | --- |
| **Primers for qPCR** | | |
| CCND1 | AATGTGTGCAGAAGGAGGTC | GGGATGGTCTCCTTCATCTT |
| IL-8 | tcagagacagcagagcacac | ttagcactccttggcaaaac |
| β-actin | ACTGGAACGGTGAAGGTGAC | AGAGAAGTGGGGTGGCTTTT |
| **Primers for ChIP** | | |
| miR-101 | TTAAGGCAACAGCACTCCCTTTGTTGAAAC | TTTCATGAGCCCTGTGAGTCTTCTGATAAC |
| MMP1 | CCTCTTGCTGCTCCAATATC | TCTGCTAGGAGTCACCATTTC |
| Neg Ctrl | CTGTTCTCTGCATGGCTGTC | ACCTTATTCCAGCTCCTCGG |
| **Primers for EMSA (Mutant sites were underlined)** | | |
| SiteA | TCCCACTGACTCATTGGAAGAGCTGTGTGACTCAGTGTAT | ATACACTGAGTCACACAGCTCTTCCAATGAGTCAGTGGGA |
| SiteA mutant oligo | TCCCACCAGTCTGTTGGAAGAGCTGTGCAGTCTGGTGTAT | ATACACCAGACTGCACAGCTCTTCCAACAGACTGGTGGGA |
| SiteB | TGTATGTGTGTCACATTTGAAGTCACCAGTT | AACTGGTGACTTCAAATGTGACACACATACA |
| SiteB mutant oligo | TGTATGCACACTGCATCCAGGACTGCCAGTT | AACTGGCAGTCCTGGATGCAGTGTGCATACA |
|  |  |  |
